# Intermittent theta burst stimulation to the left dorsolateral prefrontal cortex improves cognitive function in polydrug use disorder patients: a randomized controlled trial

**DOI:** 10.3389/fpsyt.2023.1156149

**Published:** 2023-05-25

**Authors:** Ling Dong, Wen-Cai Chen, Hang Su, Mei-Ling Wang, Cong Du, Xing-ren Jiang, Shu-fang Mei, Si-Jing Chen, Xiu-Jun Liu, Xue-Bing Liu

**Affiliations:** ^1^Wuhan Mental Health Center, Wuhan, Hubei Province, China; ^2^Wuhan Hospital for Psychotherapy, Wuhan, Hubei Province, China; ^3^Shanghai Mental Health Center, Shanghai, China

**Keywords:** polydrug use disorders, iTBS, cognitive function, GABA-Aα5, IL-10

## Abstract

**Background:**

Polydrug abuse is common among opioid users. Individuals who use both heroin and methamphetamine (MA) have been shown to experience a wide range of cognitive deficits. Previous research shows that repetitive transcranial magnetic stimulation (rTMS) can change cerebral cortical excitability and regulate neurotransmitter concentration, which could improve cognitive function in drug addiction. However, the stimulation time, location, and possible mechanisms of rTMS are uncertain.

**Methods:**

56 patients with polydrug use disorder were randomized to receive 20 sessions of 10 Hz rTMS (*n* = 19), iTBS (*n* = 19), or sham iTBS (*n* = 18) to the left DLPFC. All patients used MA and heroin concurrently. Cognitive function was assessed and several related proteins including EPI, GABA-Aα5, IL-10, etc. were quantified by ELISA before and after the treatment.

**Results:**

Baseline RBANS scores were lower than normal for age (77.25; IQR 71.5–85.5). After 20 treatment sessions, in the iTBS group, the RBANS score increased by 11.95 (95% CI 0.02–13.90, *p* = 0.05). In particular, there were improvements in memory and attention as well as social cognition. Following treatment, serum EPI and GABA-Aα5 were reduced and IL-10 was elevated. The improvement of immediate memory was negatively correlated with GABA-Aα5 (*r* = −0.646, *p* = 0.017), and attention was positively correlated with IL-10 (*r* = 0.610, *p* = 0.027). In the 10 Hz rTMS group, the improvement of the RBANS total score (80.21 ± 14.08 before vs.84.32 ± 13.80 after) and immediate memory (74.53 ± 16.65 before vs.77.53 ± 17.78 after) was statistically significant compared with the baseline (*p <* 0.05). However, compared with the iTBS group, the improvement was small and the difference was statistically significant. There was no statistically significant change in the sham group (78.00 ± 12.91 before vs.79.89 ± 10.92 after; *p >* 0.05).

**Conclusion:**

Intermittent theta burst stimulation to the left DLPFC may improve cognitive function in polydrug use disorder patients. Its efficacy appears to be better than that of 10 Hz rTMS. The improvement of cognitive function may be related to GABA-Aα5 and IL-10. Our findings preliminarily demonstrate the clinical value of iTBS to the DLPFC to augment neurocognitive recovery in polydrug use disorders.

## 1. Introduction

Around 275 million people used drugs in 2021 globally; 13% or 36.3 million people had a drug use disorder (1). In China, around 1.458 million people use heroin and other opioids, accounting for 49.3% of the total number of registered drug users. In addition, 1.459 million people used synthetic drugs, including 1.19 million who used methamphetamine, an increase of 40.5% over the previous year (2). As neurotoxic substances, drugs have long been known to cause cognitive deficits. A meta-analysis showed impairment in most cognitive areas of methamphetamine use disorder (MUD), including memory, attention, executive function, verbal/verbal fluency, and social cognition (3). The rate of polydrug use disorders (multiple simultaneous drug use) has been significantly increasing over the past 2 decades (4). Epidemiological studies have reported a high prevalence of polydrug use (30–49.7%), particularly among individuals with opioid use disorder. Chronic polydrug abusers show multiple cognitive impairments (5). Compared to alcohol use disorder, polydrug users show trends for worse performance in general intelligence, auditory-verbal learning, and decision-making tasks (6).

Transcranial magnetic stimulation (TMS) is a noninvasive technique that allows electrical currents to be induced in focal areas of the cerebral cortex, which can stimulate local and related distal cortical and subcortical areas, induce excitatory changes, and the changes can persist for around an hour following the stimulation (7). rTMS has become an evidence-based therapy for treatment-resistant and endorsed by several Clinical Practice Guidelines to be used in the treatment of mental and substance use disorders. Theta burst stimulation (TBS) is a special highly efficient rTMS sequence (8, 9). TBS has two variants: intermittent theta burst stimulation (iTBS) that improves cortical excitability, and continuous theta burst stimulation (cTBS) that reduces excitability. In previous studies, 10 Hz rTMS has been often used to treat substance dependent patients, Su et al. reported that five sessions of 10 Hz rTMS over the DLPFC could improve verbal learning and memory and social–emotional cognition in MA-addicted subjects (10), It has been shown to significantly reduce craving, and improve emotional problems and cognitive function (11). In addition, multiple-day rTMS improved arbitrary face-word pairings memory and hippocampal-cortical functional connectivity (12). In recent years, there have been many studies of iTBS for substance addiction. Previous work suggests that attention bias and beta oscillation during the attentional processing of words in patients with MUD can be modulated by iTBS applied to the left DLPFC, and iTBS significantly reduces craving and improves cognition and sleep quality for methamphetamine addicts (13). The iTBS treatment protocol positively affects behavioral inhibition in patients with heroin addiction (14). 10 Hz rTMS takes around 20 min to complete 900 pulses; iTBS is more efficiently, which takes only 4 min and 52 s to complete the same pulses. Compared with 10 Hz rTMS, TBS maybe more acceptable to patients because of its short treatment time. Even both 10 Hz rTMS and iTBS had similar results of improving cognition in patients with substance addiction, there have no studies to compare the difference between them. So our study was designed to validate previous results and initially compare the efficacy of iTBS with 10 Hz rTMS.

There has been no effective medical treatment for polydrug use disorders. Therefore, novel treatment approaches for polydrug use disorders are desperately needed. Considering the prefrontal dysfunction and cognitive impairments that have been observed in patients with polydrug use disorders and the effectiveness of rTMS for other psychiatric diseases, we hypothesize that iTBS to the DLPFC may also improve the cognitive function of polydrug users. We tested iTBS to the DLPFC as our therapeutic approach. In short, the purpose of this study was to test whether iTBS to the left DLPFC would modulate cognitive function in polydrug use disorders by utilizing a randomized, double-blind, sham-controlled study design. We tested whether cognitive function would be influenced by using a detailed CogState Battery of standardized neuropsychological tasks: The Repeatable Battery for the Assessment of all the Neuropsychological States (RBANS). In addition, we measured several related blood indicators, including epinephrine (EPI), gamma aminobutyric acid receptor Aα5 (GABA-Aα5), interleukin10 (IL-10), and others, to probe the mechanisms involved in cognitive function in these patients.

## 2. Materials and methods

### 2.1. Participant selection

We recruited 56 patients with polydrug use disorder from The First Health Clinic Center for Addiction of Wuhan Mental Health Center, Wuhan, China. Participants were treated with a real or sham iTBS or 10 Hz rTMS protocol from 2019.07 to 2021.10 utilizing a randomized, double-blind, sham-controlled study design. On the day of the clinical intake, patients provided written informed consent. The approval for the protocol was obtained from the Ethical Committee for the Psychological Research of the hospital (Wuhan Mental Health Center). This clinical trial was registered based on the ICMJE guidelines with a clinical trial ID: NCT04264741. Participants were aged between 40 and 62 years and met the diagnostic criteria for heroin and MA use disorder (confirmed by a double positive urine test) according to the Diagnostic and Statistical Manual of Mental Disorders-5 (DSM-5), as assessed by a clinical psychiatrist specialized in substance use disorders (SUDs). During the study, we did not impose any restrictions on the drugs used; we simply observed and recorded the results.

### 2.2. Clinical measures

The participants were interviewed by trained investigators using a detailed questionnaire including general information, social demographic characteristics, current and prior substance use behaviors, and medical and psychological history.

### 2.3. Blood sampling and serum measurements

We collected serum samples from 37 participants before the first session of iTBS and after the entire treatment course with real or sham iTBS. The serum was separated, aliquoted, and stored at −80°C until analysis. The blood marker levels were measured by a commercial sandwich enzyme-linked immunosorbent assay (ELISA; Beijing rongxinzhihe Biotechnology Co., Ltd., Wuhan, China) according to the manufacturer’s instructions. First, the 10 μL serum samples and 40 μL sample diluent were mixed in each well. Then, 50 μL sample diluent was added to the standard well. After that, 100 μL of HRP-conjugate reagent was added to each well, covered with an adhesive strip, and incubated for 60 min at 37°C. The wells were washed and then incubated with HRP Conjugate working solution for 30 min, again washed and incubated with a substrate reagent for 15 min at 37°C and finally the stop solution was added. The Optical Density (O.D.) was read at 450 nm using a microtiter plate reader within 15 min.

The participant ID was coded and the real number IDs were retained by the principal investigator until all biochemical analyses were completed. Inter- and intra-assay variation coefficients were 8 and 5%, respectively.

### 2.4. Cognitive assessment

All participants were administered the RBANS before and after all treatments with rTMS, to measure cognitive function (15). RBANS is designed to assess and characterize cognitive function over time and covers a broad range of difficulties to minimize floor and ceiling effects. RBANS includes five domains, with scores ranging from 40 to 16i for which age-specific normative data are available; these were generated in a Scandinavian population cohort scoring a mean of 100 (standard deviation (SD) ± 15). The five domains are immediate memory, visuospatial function, language, attention, and delayed memory. The RBANS includes 12 sub-tests that are used to calculate five age-adjusted index scores and then a total score and an emotional identification task. The five test indices comprise immediate memory (story memory and list learning sub-test line orientation), visuospatial/constructional (line orientation and figure copy sub-test), language (semantic fluency and picture naming sub-tests), and delayed memory [figure recall, story recall, list recall, and recognition sub-tests, attention (coding and digit span sub-tests), and delayed memory (including figure recall, story recall, list recall, and recognition sub-tests)]. In the eyes-reading test, participants were asked to interpret the emotion associated with 30 images of eyes, which reflects an individual’s social cognition. The RBANS was previously translated into Chinese, although Chinese normative data do not exist. Attempts have been made to establish a minimal clinically important difference using an anchor-based approach but without solid results. Therefore, we chose not to use a specific cut-off value for clinical relevance.

### 2.5. rTMS treatment

**Figure 1:**
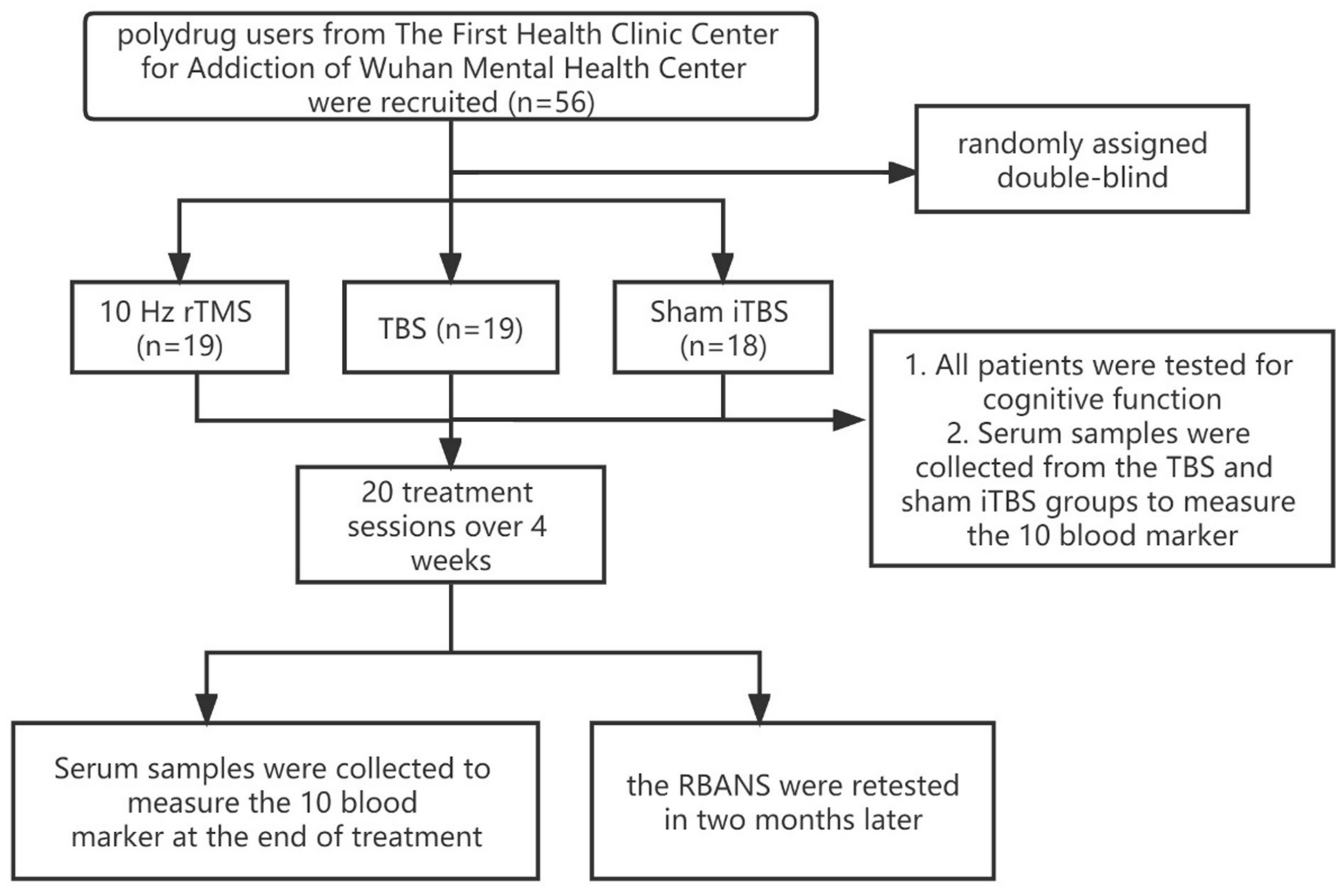
CONSORT flowchart of the study.

Fifty-six participants were randomly assigned into a 10 Hz rTMS group (*n* = 19), an iTBS group (*n* = 19), or a sham iTBS group (*n* = 18) by a researcher who was not involved in the assessment used the random number table method. The rTMS stimulation protocol was administered by a trained clinical physiologist. The patient’s motor threshold (MT) was confirmed through the left motor cortex, the lowest intensity which can produced a motor response was found in the right abductor pollicis brevis muscles (APB), that could produce motor-evoked responses of at least 50 mV five in 10 trials. During the treatment, the coil was placed over the left prefrontal area at a point 5 cm anterior to the scalp position where the motor threshold was determined. During treatment, patients reclined on a comfortable bed while TMS stimuli were delivered to the left DLPFC. The stimulator device was a CCY-I TMS instrument with a Cool-B80 butterfly coil (Yiruide Co., Wuhan, China). By reviewing relevant literature, about the use of 10 Hz rTMS, there was stimulation for 1.5 s with intervals of 58.5 s (16) and stimulation for 4 s with intervals of 26 s (17). In our study, we aimed to compare the efficacy of the 10 Hz rTMS and the iTBS; hence, the same number of pulses (900 pulses) was used. After discussion and calculation, we determined the parameters of the 10 Hz, which was 10 Hz, 80–100%MT, 2 s on and 18 s off for 15 min, with 900 pulses per day (18). The iTBS applied 3 × 50 Hz pulses in 5 Hz packets over 2 s, followed by 8 s of inactivity, with repeated cycles that continued for 292 s, 900 pulses per day, 80–100% of MT. The total treatment time was around 5 min. In the sham iTBS group, patients received a similar pseudo-stimulation treatment. The treatment site and method were the same as the real stimulation, but the coil was placed at 90° vertical during the stimulation; a treatment-like sound was generated but the stimulation is weak and cannot penetrate the skull. Patients underwent one treatment session per day from Monday to Friday for 4 weeks. Adverse events were self-reported and collected. Each patient was evaluated by the psychiatrist and provided the appropriate dose of methadone (see CONSORT flowchart in Figure 1).

### 2.6. Statistical analyses

SPSS (version 21.0) was used for all statistical analyses. The demographic and clinical variables of all participants were compared using ANOVA for continuous variables and *X*^2^ for categorical variables. We compared the difference between groups at baseline, after the intervention, and before and after the intervention. When the data were normally distributed, ANOVA or *t*-test was employed. Changes in cognitive function were calculated by subtracting the post-treatment score from the pre-treatment score. When the data were not normally distributed, nonparametric tests were used. To compare baseline and post-intervention, paired *t*-tests were utilized. Pearson correlation coefficients were calculated to analyze the correlation between cognitive function and biochemical markers in the blood. Data are presented as mean ± SD. All tests were two-tailed with the significance threshold set at 0.05. When making multiple comparisons, we corrected the *p* value (P/n) according to the number of comparisons.

## 3. Results

### 3.1. Demographic and drug use information

Demographic characteristics and drug use summary data are shown in Table 1. There were no differences between the 10 Hz rTMS group or iTBS or sham iTBS group in terms of age, education years, marriage, employment, or body mass index (BMI). No differences were found between the three groups in terms of onset age, abstinence period, duration of MA use, or the MA dose and frequency. All of the patients continued to use drugs throughout the study (Table 1).

**Table 1:** Demographic and clinical characteristics of the participants.

	10 Hz rTMS (*n* = 19)	iTBS (*n* = 19)	Sham iTBS (*n* = 18)	*F*/*χ*^2^	*p*
Age (years)	49.35 ± 1.86	49.89 ± 5.40	48.78 ± 6.32	1.093	0.566
Gender (male/female)	16/3	15/4	16/2	0.679	0.712
Education (years)	9.62 ± 1.27	9.83 ± 1.38	9.56 ± 1.54	0.060	0.573
Marriage				1.544	0.819
Married	8 (42%)	8 (42%)	8 (44.4%)		
Divorced	7 (36.8%)	6 (31.6%)	8 (44.4%)		
Other(including widowed and never have marriage)	4 (21.1%)	5 (26.3%)	2 (11.2%)		
Employment(yes/no)	5/14	6/13	6/12	0.236	0.889
BMI	21.56 ± 4.13	21.69 ± 3.67	21.84 ± 4.55	0.032	0.912
Age at first experience (years)	41.32 ± 7.61	40.47 ± 5.60	42.61 ± 8.19	3.275	0.358
Accumulated use time(years)	8.36 ± 4.17	9.26 ± 3.91	7.22 ± 5.42	5.264	0.196
Baseline					
Dose of heroin use per day (g)	1.02 ± 2.15	1.14 ± 2.26	0.85 ± 1.21	1.029	0.633
Frequency of drug use 30 days before baseline				7.042	0.317
Daily	8 (42.11%)	10 (52.63%)	5 (27.78%)		
3-5times a week	7 (36.84%)	4 (21.05%)	8 (44.45%)		
Once a week	2 (10.53%)	4 (21.05%)	1 (5.56%)		
1–3 times a month	2 (10.53%)	1 (5.26%)	4 (22.22%)		
After 20 treatment sessions over 4 weeks					
Dose of heroin use per day (g)	1.01 ± 1.73	1.12 ± 1.68	1.05 ± 1.36	1.542	0.807
Frequency of drug use during 4 weeks of intervention				5.41	0.493
Daily	7 (36.84%)	9 (47.37%)	5 (27.78%)		
3–5times a week	7 (36.84%)	3 (15.79%)	7 (38.89%)		
Once a week	3 (15.79%)	5 (26.32%)	2 (11.11%)		
1–3 times in all	2 (10.53%)	2 (10.53%)	4 (22.22%)		

Data are presented as mean (SD) unless otherwise specified.

### 3.2. The effect of iTBS/rTMS on cognition

**Figure 2:**
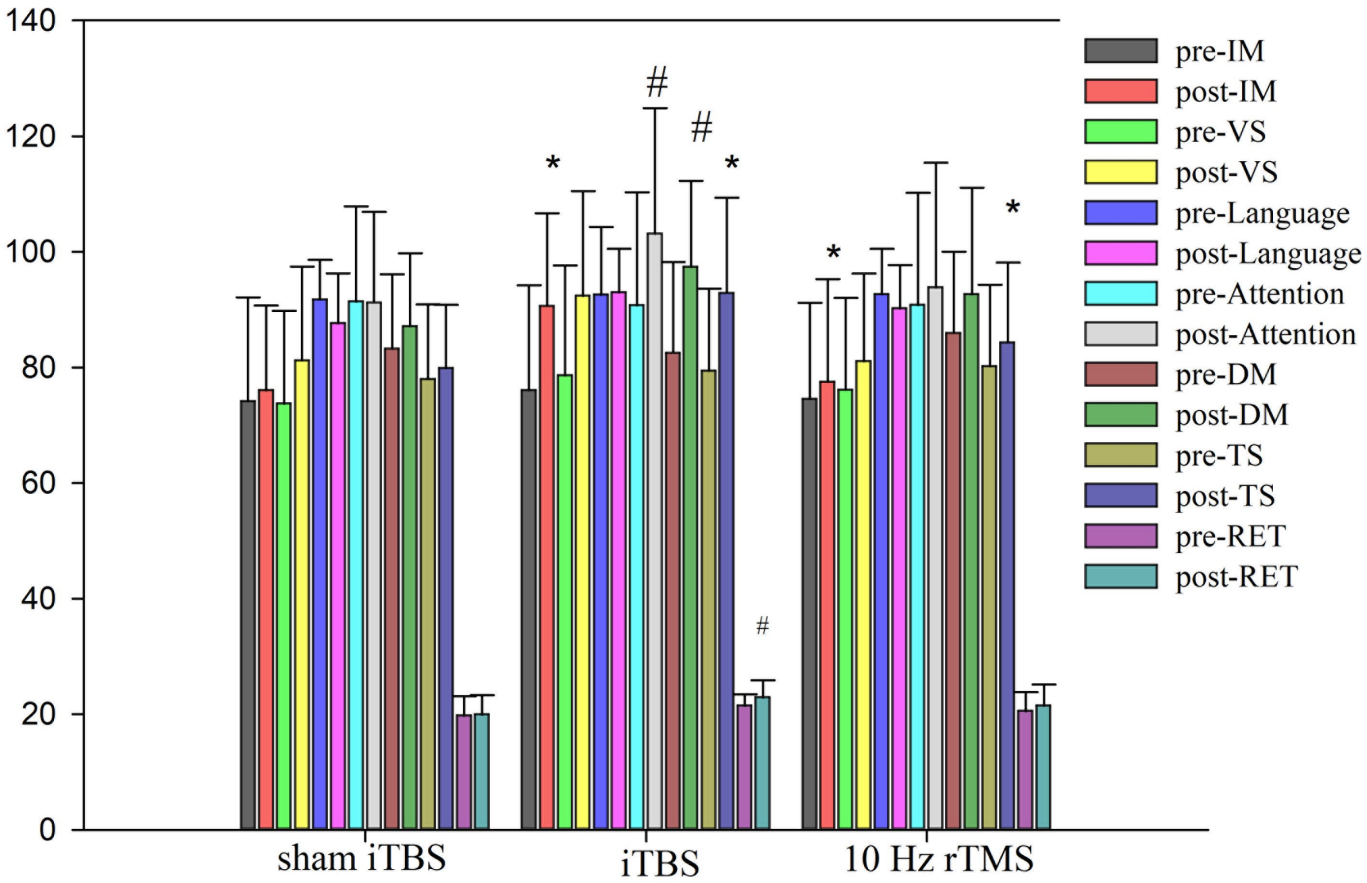
Cognitive function scores pre and post intervention. ANOVA was used, • means *p* < 0.05. ^#^Compared with the baseline, group have statistical difference. Immediate memory (IM, story memory and list learning sub-test line orientation), visual spatial/constructional (VS, line orientation and figure copy sub-test), language (semantic fluency and picturenaming sub-tests), attention (including coding and digit span sub-tests), delayed memory (DM, including figure recall, story recall, list recall, and recognition sub-tests), Rbans total score (TS), and Read the eye test (RET).

At baseline, there were no differences in the total RBANS score between groups (80.21 ± 14.08 in traditional rTMS and 79.42 ± 14.22 in iTBS group and 78.00 ± 12.91 in sham iTBS group; *p* > 0.05), and there were also no differences in all indices between the three groups (*p* > 0.05; Table 2). After 20 treatment sessions over 4 weeks, compared with any other group, the iTBS group showed improvements in the RBANS total score, and immediate memory (*p* < 0.05). Compared with the baseline, iTBS showed a significant improvement in the RBANS score, immediate memory, visual spatial, attention, delayed memory, and read-the-eye test (*p* < 0.05). The 10 Hz rTMS showed a significant improvement in the RBANS score and immediate memory (*p* < 0.05). Compared with the sham group, the D-value of iTBS showed a significant improvement in the RBANS score, immediate memory, attention, and read-the-eye test (*p* < 0.017). Compared with the 10 Hz rTMS group, the D-value of iTBS showed a significant difference in the RBANS score and immediate memory (*p* < 0.017). In the sham iTBS group, no significant difference was found before and after the treatment (*p* > 0.05; Figure 2).

**Table 2:** Cognitive function scores before and after intervention.

Outcomes	Baseline			Post-treat			Difference value(median)			*p* value		
	Sham iTBS	iTBS	10 Hz rTMS	Sham iTBS	iTBS	10 Hz rTMS	Sham iTBS	iTBS	10 Hz rTMS	Baseline	Post-treat	Diff
Rbans total score	78.00 ± 12.91	79.42 ± 14.22	80.21 ± 14.08	79.89 ± 10.92	92.90 ± 16.46#	84.32 ± 13.80#	3.5	13^&^	4^&^	0.885	0.021*	0.039*
Immediate memory	74.17 ± 17.96	76.05 ± 18.19	74.53 ± 16.65	76.06 ± 14.65	90.63 ± 16.02#	77.53 ± 17.78#	−2.5	15^&^	2^&^	0.941	0.015*	0.012*
Visual spatial	73.72 ± 16.07	78.63 ± 18.98	76.11 ± 15.92	81.22 ± 16.24	92.37 ± 18.14#	81.05 ± 15.19	1.5	8	5	0.684	0.065	0.792
Language	91.72 ± 6.88	92.58 ± 11.71	92.63 ± 7.87	87.61 ± 8.65	92.95 ± 7.54	90.21 ± 7.50	−4.5	0	−3	0.944	0.131	0.688
Attention	91.39 ± 16.45	90.74 ± 19.55	90.79 ± 19.42	91.22 ± 15.67	103.11 ± 21.74#	93.84 ± 21.57	0	10^&^	3	0.993	0.017*	0.007*
Delayed memory	83.22 ± 12.90	82.53 ± 15.67	85.95 ± 14.07	87.11 ± 12.65	97.37 ± 14.92#	92.63 ± 18.45	2	14	4	0.74	0.144	0.083*
Read the eye test	19.72 ± 3.36	21.42 ± 2.04	20.47 ± 3.34	19.89 ± 3.41	22.84 ± 3.00#	21.42 ± 3.69	−0.5	2^&^	0	0.227	0.256	0.007*

ANOVA was used,^*^means *p* < 0.05.

Pair T test was used, ^#^means that compared with the baseline, the group has statistical difference.

Non-parametric tests were used, corrected *p* was 0.17 = 0.05/3, and ^&^ means that compared with the sham group, the group has statistical difference.

Immediate memory (story memory and list learning sub-test line orientation), visual spatial/constructional (line orientation and figure copy sub-test), language (semantic fluency and picture naming sub-tests), attention (including coding and digit span sub-tests), and delayed memory (including figure recall, story recall, list recall, and recognition sub-tests).

### 3.3. Serum biochemical levels/changes in blood biochemical indices

**Figure 3:**
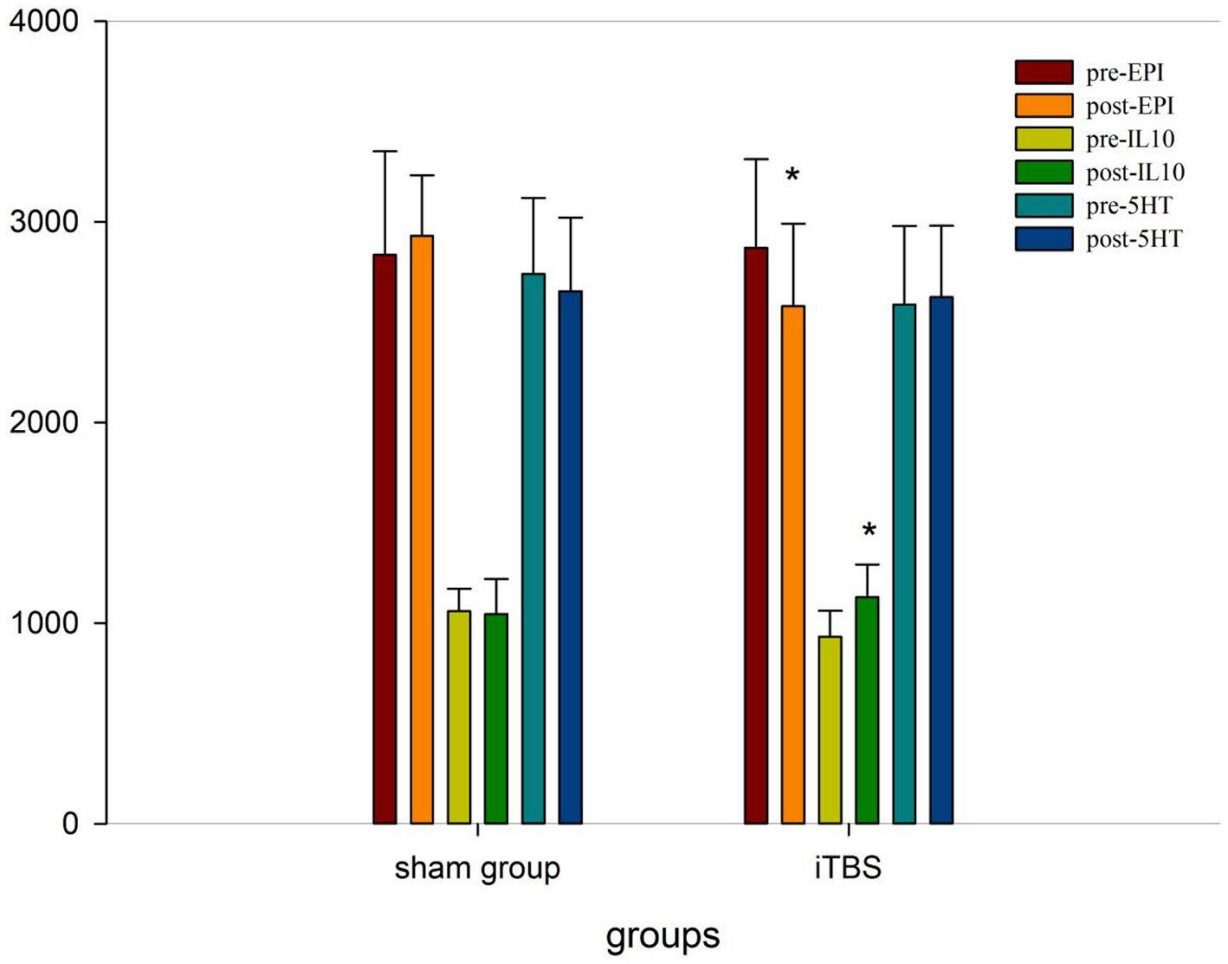
Blood indicators pre and post iTBS intervention.

**Figure 4:**
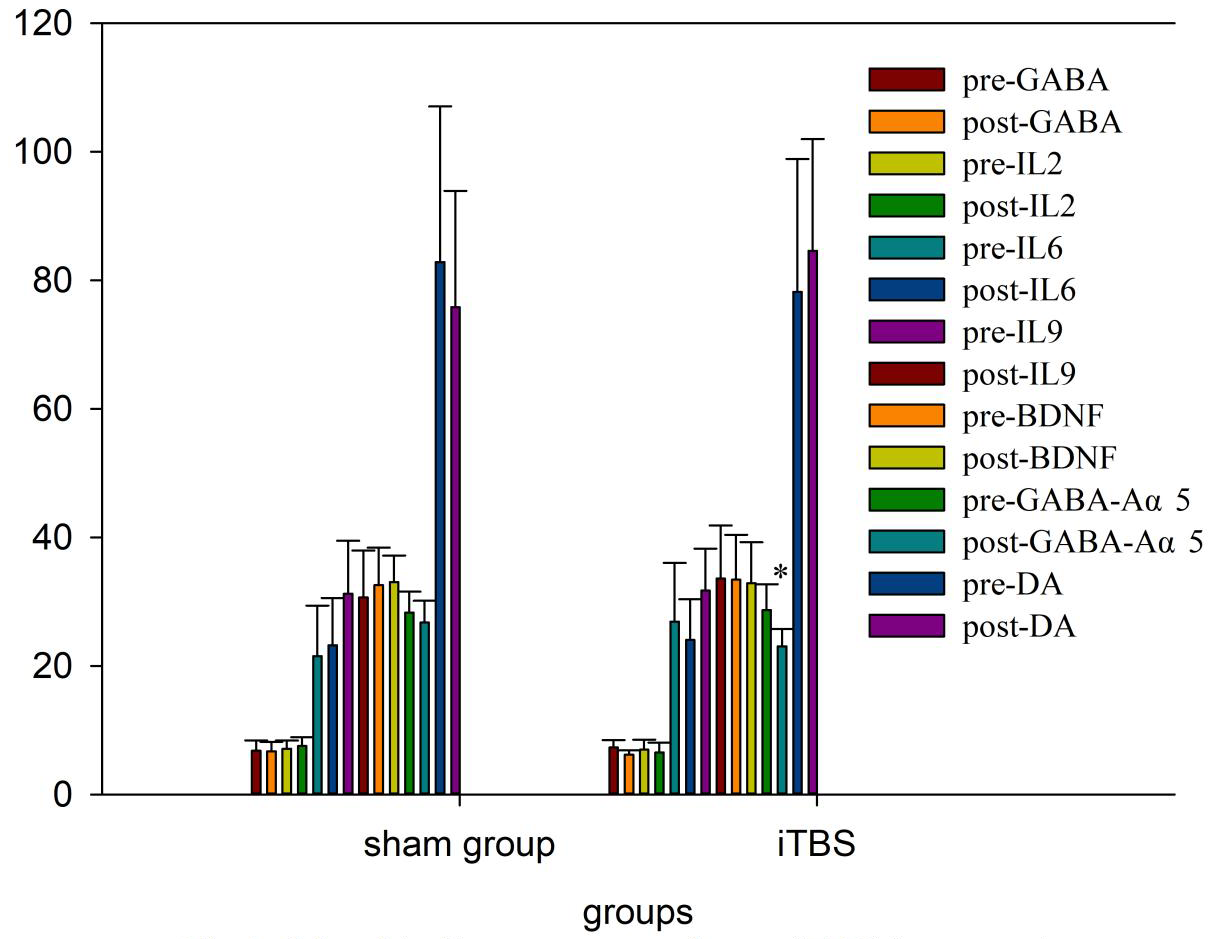
Blood indicators pre and post iTBS intervention.

We tested the concentrations of 10 blood markers. Before treatment, there were no differences in any of the markers, except for IL-10, between the iTBS and sham iTBS groups (*p* > 0.05; Table 3). Compared to baseline, after 20 treatment sessions over 4 weeks, the iTBS group showed lower epinephrine (EPI; 2870.43 ± 441.7 vs. 2578.9 ± 412.13), higher interleukin 10 (IL-10; 931.57 ± 128.68 vs. 1128.94 ± 162.86), and lower γ -aminobutyric acid Aα5 type (GABA-Aα5; 28.65 ± 4.05 vs. 23.05 ± 2.69; Figures 3, 4).

**Table 3:** Blood indicators before and after real or sham iTBS intervention.

Blood indicators	Baseline		Post-treat		Difference value (median)		*p* value			
	Sham group	iTBS	Sham group	iTBS	Sham group	iTBS	Baseline	Post-treat	iTBS paired T	Diff
EPI	2835.64 ± 515.47	2870.43 ± 441.70	2930.07 ± 301.31	2578.9 ± 412.13#	60.04	−416.27&	0.829	0.006*	0.003*	0.010*
IL-10	1059.06 ± 112.02	931.57 ± 128.68	1045.36 ± 174.25	1128.94 ± 162.86#	−77.48	154.91&	0.003*	0.146	0.000*	0.002*
GABA-Aα5	28.28 ± 3.26	28.65 ± 4.05	26.73 ± 3.4	23.05 ± 2.69#	−2.15	−6.93&	0.764	0.001*	0.000*	0.004*
DA	82.81 ± 24.22	78.2 ± 20.68	75.81 ± 18.08	84.58 ± 17.37	−0.13	0.08	0.613	0.239	0.312	0.214
5-HT	2741.03 ± 377.9	2586.52 ± 392.3	2653.49 ± 366.87	2623.94 ± 355.87	−34.48	20.33	0.29	0.841	0.798	0.597
BDNF	32.58 ± 5.82	33.4 ± 6.98	33.04 ± 4.14	32.88 ± 6.35	0.05	0.12	0.733	0.943	0.953	0.368
IL-2	7.08 ± 1.3	6.97 ± 1.54	7.55 ± 1.33	6.52 ± 1.53	−0.04	−0.23	0.841	0.089	0.416	0.291
IL-6	21.53 ± 7.84	26.89 ± 9.13	23.23 ± 7.33	24.04 ± 6.31	−1.09	−2.05	0.103	0.768	0.099	0.093
IL-9	31.24 ± 8.22	31.7 ± 6.54	30.66 ± 7.3	33.59 ± 8.25	−1.44	−1.35	0.868	0.363	0.669	0.351
GABA	6.83 ± 1.58	7.29 ± 1.16	6.72 ± 1.45	6.18 ± 0.71	−0.11	−0.34	0.370	0.234	0.052	0.153

^*^p < 0.05.

# Compared with the baseline, the group has statistical difference.

& Compared with the sham group, the group has statistical difference.

### 3.4. Relationship between cognitive function and rTMS treatment and serum biochemical levels

Pearson correlation analyses showed that immediate memory was negatively correlated with GABA-Aα5 (*r* = −0.646, *p* = 0.017) and attention was positively correlated with IL-10 (*r* = 0.610, *p* = 0.027) in the iTBS group (Table 4). However, no significant correlations were detected between other indices of RBANS and other serum biochemical levels (Table 4).

**Table 4:** Pearson correlation analysis of cognitive functioning and blood indicators.

	GABA-Aα5		IL-10	
	*r*	*p*	*r*	*p*
Immediate memory	−0.646	0.017	0.390	0.188
attention	0.377	0.204	0.610	0.027

r, Pearson correlation coefficient.

## 4. Discussion

In our study, we found that iTBS to the left DLPFC significantly improved cognitive function, including memory and attention, and social cognition in heroin and MA-addicted individuals. These results are consistent with previous studies (13, 19). However, another study suggested inconsistent results. For instance, Turriziani’s study showed that iTBS to the right DLPFC led to a deterioration in memory performance while iTBS to the left DLPFC had no effect on recognition memory performance in healthy controls (20). Previous research has indicated that the left and right DLPFC serve different functions in memory tasks (21). The inconsistency of previous findings may be attributed to differences in the location of stimulation. Our study found that applying 10 Hz rTMS over the DLPFC can enhance memory in polydrug users, which is consistent with previous research (10). Furthermore, our results suggest that iTBS improves immediate memory to a greater extent than 10 Hz rTMS. Other studies have demonstrated that the frequency of stimulation is the primary factor that determines the direction of excitability modulation (22). Higher frequency iTBS (50 Hz) can increase neuronal activity and cortical excitability, which in turn can improve mood. One clinical study reported that iTBS was more effective than 10 Hz rTMS in alleviating depression (23), and a better mood might contribute to the greater improvement in cognitive function. To date, this is the first trial to investigate the comparative influence of iTBS and 10 Hz rTMS on cognitive function in polydrug users. Compared with 10 Hz rTMS, iTBS takes less time and the effect may be better, so it is more likely to be accepted by patients. We found significantly lower cognitive function levels in adults with polydrug use disorders. Using normative data as a reference, polydrug use disorder patients performed significantly worse across all five RBANS indices, with attention and memory and social cognition showing the largest differences. These findings are in agreement with previous studies showing reduced cognitive function in patients who use methamphetamine and heroin (24).

In our study, GABA-Aα5 was significantly reduced and was significantly associated with improved memory function in the iTBS group. Experiments in animals and humans have elucidated some of the potential mechanisms of rTMS in improving cognitive function. Zheng et al. found the increase of GABA-Aα5 might be involved in learning-memory dysfunction in the offspring of chronic ethanol-treated rats and that a GABA-Aα5 inhibitor could be an effective way to treat alcohol-induced cognitive impairment (25). Prévot et al. found that reduced signaling of the GABA-Aα5 receptor can improve cognitive function (26). We found that iTBS could act as a GABA-Aα5 inhibitor, which led to a decrease in GABA-Aα5 levels and an improvement in memory in our study. This is consistent with the results of previous studies. Su et al. found that the effect of rTMS on cognitive function in individuals with methamphetamine dependence may be related to changes in GABA levels in the prefrontal cortex (27).

Inflammation has been shown to play an important role in the cognitive deficits of polydrug use disorders. MA causes an increase in pro-inflammatory cytokines in animal models and humans. Heroin addiction also influences the immune system. Kobeissy et al. showed that three major cytokines (IL-1β, IL-6, and IL-10) were elevated in an MA group compared to a saline group (28). Kohno et al. found that IL-6 levels are higher in MA users than in controls (29). Mice subcutaneously administered heroin showed an increased production of IL-1β, IFN-γ, and IL-12 within 2 h, but the production of the anti-inflammatory cytokines IL-4 and IL-10 was decreased (30). In our study, IL-2, IL-6, and IL-9 levels were not changed by iTBS or sham stimulation, but IL-10 was significantly increased and was significantly associated with improved attention in the iTBS group. This suggests that iTBS may modulate immunity by altering IL-10 expression, and thus improve cognitive function. This is consistent with previous studies, Zhang et al. found that cognitive impairment could be alleviated by IL-10 (31). where IL-10 was reported to be increased after rTMS treatment in middle cerebral artery occlusion model rats, which significantly reduced neuronal apoptosis, promoted neuronal plasticity, and improved cognitive function (32). Multiplex cytokine bioassays showed that iTBS increased IL-10 in injury mice with middle cerebral artery occlusion (33). In addition, our study found a significant decline in EPI after treatment but did not find a correlation between this change and cognitive function. This is inconsistent with relevant research, Jiang et al. found a relationship between sevoflurane-induced cognitive impairment and EPI adrenoceptors in the cerebral cortex of rats (34).

Our study has some limitations. First, participants received only 20 sessions of stimulation without subsequent treatments. Secondly, to avoid time effects on cognitive function, especially for memory, we remeasured cognitive function 2 months after the end of treatment. However, blood was drawn immediately after treatment. This discrepancy in timings may affect the correlation between changes in cognitive function and those blood markers, although it does suggest that early biomarker responses are associated with later cognitive status. Future studies may consider continued rTMS treatment 2–3 times a week after the first 20 sessions, with continued cognitive function tests and longitudinal blood analyses, to better model the relationship between changes in cognitive function and blood indicators, and observe the duration of rTMS effects. In addition, we did not assess depression and anxiety symptoms, which is a related oversight because mood, anxiety, and other psychological symptoms are over represented in drug users. The effective treatment of emotional symptoms by iTBS/10 Hz rTMS may be attributed to improved cognitive abilities. We will focus on emotional symptoms in a follow-up study.

In conclusion, we preliminary found that iTBS to the left DLPFC could improve cognitive function in polydrug use disorder patients, and its efficacy was superior to that of 10 Hz rTMS. At the same time, serum GABA-Aα5 and EPI levels decreased and IL-10 increased. These findings suggest that iTBS-rTMS may act as a GABA-Aα5 inhibitor and IL-10 agonist. Future studies should focus on related proteins change and functional MRI of the brain to identify the mechanisms of iTBS, and ideally conduct longer treatment and follow-up.

## Data availability statement

The original contributions presented in the study are included in the article/supplementary materials, further inquiries can be directed to the corresponding authors.

## Ethics statement

The studies involving human participants were reviewed and approved by Ethics Committee of Wuhan Mental Health Center. The patients/participants provided their written informed consent to participate in this study.

## Author contributions

LD was responsible for the study concept and design and did the rTMS intervention and drafted the manuscript. X-JL, X-BL, and S-JC helped design the study. M-LW, CD, X-rJ, and S-fM acquired the clinical data. W-CC conducted the data analysis. LD, W-CC, HS, M-LW, CD, X-rJ, S-fM, S-JC, X-JL, and X-BL provided critical revision of the manuscript for important intellectual content. All authors contributed to the article and approved the submitted version.

## Funding

This study was supported by the Key Projects for Youth of Wuhan Health and Family Planning Commission (WX19Q23 to LD, 2019); National Nature Science Foundation (82130041); and Shanghai Municipal Science and Technology Major Project (2018SHZDZX05). The authors gratefully acknowledge the financial support from the above program. The sponsor had no role in the study design, data collection, analysis, interpretation of the results, or writing of the report.

## Conflict of interest

The authors declare that the research was conducted in the absence of any commercial or financial relationships that could be construed as a potential conflict of interest.

## Publisher’s note

All claims expressed in this article are solely those of the authors and do not necessarily represent those of their affiliated organizations, or those of the publisher, the editors and the reviewers. Any product that may be evaluated in this article, or claim that may be made by its manufacturer, is not guaranteed or endorsed by the publisher.
